# High-Resolution Geospatial Database: National Criteria-Air-Pollutant Concentrations in the Contiguous U.S., 2016–2020

**DOI:** 10.1002/gdj3.70005

**Published:** 2025-04-07

**Authors:** Tianjun Lu, Sun-Young Kim, Julian D. Marshall

**Affiliations:** 1 Department of Epidemiology and Environmental Health, College of Public Health, University of Kentucky, Lexington, Kentucky, USA; 2 Department of Cancer AI and Digital Health, Graduate School of Cancer Science and Policy, National Cancer Center, Goyang-si, Gyeonggi-do, Korea; 3 Department of Civil and Environmental Engineering, University of Washington, Seattle, Washington, USA

**Keywords:** criteria air pollutant, empirical model, environmental disparity, environmental hazard, exposure assessment, geospatial data, land use regression

## Abstract

Concentration estimates for ambient air pollution are used widely in fields such as environmental epidemiology, health impact assessment, urban planning, environmental equity and sustainability. This study builds on previous efforts by developing an updated high-resolution geospatial database of population-weighted annual-average concentrations for six criteria air pollutants (PM_2.5_, PM_10_, CO, NO_2_, SO_2_, O_3_) across the contiguous U.S. during a five-year period (2016–2020). We developed Land Use Regression (LUR) models within a partial-least-squares–universal kriging framework by incorporating several land use, geospatial and satellite–based predictor variables. The LUR models were validated using conventional and clustered cross-validation, with the former consistently showing superior performance in capturing the variability of air quality. Most models demonstrated reliable performance (e.g., mean squared error—based *R*^2^ > 0.8, standardised root mean squared error < 0.1). We used the best modelling approach to develop estimates by Census Block, which were then population-weighted averaged at Census Block Group, Census Tract and County geographies. Our database provides valuable insights into the dynamics of air pollution, with utility for environmental risk assessment, public health, policy and urban planning.

## 1 | Introduction

High spatial-resolution estimates for levels of ambient (outdoor) air pollution are useful for researchers and practitioners in epidemiology, public health and policy, health disparities, environmental equity, urban planning and climate change (Bonell et al. 2023; Burger et al. 2024; Clark et al. 2014; Coleman et al. 2020; Hankey et al. 2017; Hystad et al. 2011; Nadal et al. 2015; Wang et al. 2023). Databases of ambient air pollution can provide spatially precise information for identifying and analysing local pollution patterns, thereby allowing for more accurate risk assessment, air quality management and locally tailored interventions and policies (Allen and Barn 2020; Bechle et al. 2023; Colmer et al. 2020; Lu et al. 2022, 2023; Zwack et al. 2011).

Empirical models of air pollution—also called land-use regression (LUR) or Integrated Empirical Geographic (IEG) models—are a geospatial approach that can predict, with fine spatial resolution, concentrations at unmeasured locations (Amini et al. 2017; Di et al. 2019a; Hankey and Marshall 2015; Keller et al. 2015; Saha et al. 2021; Sampson et al. 2013; Van Donkelaar et al. 2019; Young et al. 2016). While details of the methods employed have evolved over time, LUR models exploit correlations between land use variables (e.g., the road network, population density, industry) and levels of ambient air pollution (Beelen et al. 2013; Hoek et al. 2008; Jerrett et al. 2005; Su et al. 2008).

Initially, LUR models were developed for single urban areas (Gilbert et al. 2005; Hankey et al. 2019; Henderson et al. 2007; Hoek et al. 2008; Jerrett et al. 2007; Lu et al. 2019; Marshall et al. 2008; Sanchez et al. 2018; Saraswat et al. 2013). Later, LUR was developed for national (Clark et al. 2014; Kerckhoffs et al. 2015; Kim et al. 2020; Novotny et al. 2011), continental (de Hoogh et al. 2016; Knibbs et al. 2016, 2018) and global scales (Larkin et al. 2023, 2017). Input variables have grown from land-use data to now include satellite-derived pollution estimates (Bechle et al. 2015; Filonchyk and Peterson 2023; Knibbs et al. 2018; Roy 2021), predictions from mechanistic models (e.g., chemical transport models) (de Hoogh et al. 2016; Goldberg et al. 2019), and microscale features (e.g., street view and point of interest) (Lu et al. 2021; Messier et al. 2018; Qi et al. 2022; Qi and Hankey 2021). Methods have shifted to better allow for non-criteria pollutants (Messier et al. 2018; Saha et al. 2022; Saraswat et al. 2013) and larger amounts of input data (i.e., more measured concentrations, more independent variables; ‘big data’ techniques) and more rapid, more sophisticated mathematical modelling (e.g., statistical approach selection, variable prioritisation, spatial coverage expansion; ‘machine learning’) (Beckerman et al. 2013; Di et al. 2019b; Goldberg et al. 2019; Hoek et al. 2015; Keller et al. 2015; Kim et al. 2020; Lu et al. 2019, 2021; Masiol et al. 2019; US EPA 2022; Van Donkelaar et al. 2019; Weichenthal et al. 2016; Wong et al. 2021).

A recent inter-comparison of spatial predictions by six national empirical models reported relatively close agreement among annual-average model predictions (Bechle et al. 2023). While the inputs and methods differed among models, the dependent data were consistent: publicly available United States Environmental Protection Agency (US EPA) regulatory monitoring station data. That result suggests that, among the approaches they compared, the actual methods might not dramatically influence the resulting annual-average predictions if the dependent variable is EPA regulatory air pollution measurements.

Here, we extend and update our previous modelling (Kim et al. 2020): national annual-average empirical models for criteria pollutants in the contiguous U.S. Specifically, in prior work we developed parsimonious national annual-average LUR models to estimate air pollutant concentrations for year-2015 and earlier. Here, we estimate concentrations for years-2016 to –2020, particularly allowing for ongoing health effect studies and exposure disparity research that would reveal evolving environmental impacts and facilitate the comparison of recent periods with historical data (Coleman et al. 2020; Lane et al. 2022; Liu et al. 2021). Prior estimates were publicly available for free download and have been integral to a range of applications (on average, currently receiving ~2 downloads per day); the same holds for the current estimates.

## 2 | Data and Methods

In general, the three steps for empirical models are (1) model building, (2) model testing, and (3) model application. Below, we describe the dependent data (pollution concentrations), the independent data (i.e., ‘predictor variables’, such as land uses and satellite data), and then the model-building, model-testing and model-application stages. The approach employed here is consistent with prior work (i.e., years 2016–2020 here; years 2015 and earlier in prior work).

### 2.1 | Dependent Data: Concentrations of Criteria Pollutants at Regulatory Monitoring Stations

For regulatory purposes, such as ensuring compliance with national standards, EPA measures ambient concentrations of criteria air pollutants at regulatory monitoring stations throughout the U.S. Here, we employ data for year-2016 to –2020 for six criteria pollutants: nitrogen dioxide (NO_2_), sulfur dioxide (SO_2_), carbon monoxide (CO), ozone (O_3_) and two types of particulate matter (PM_10_ [particles with diameters 10 μm or smaller] and PM_2.5_ [particles with diameters 2.5 μm or smaller]). Data were obtained for all Air Quality System (AQS) monitoring locations via the EPA data repository (https://www.epa.gov/outdoor-air-quality-data).

**FIGURE 1 |:**
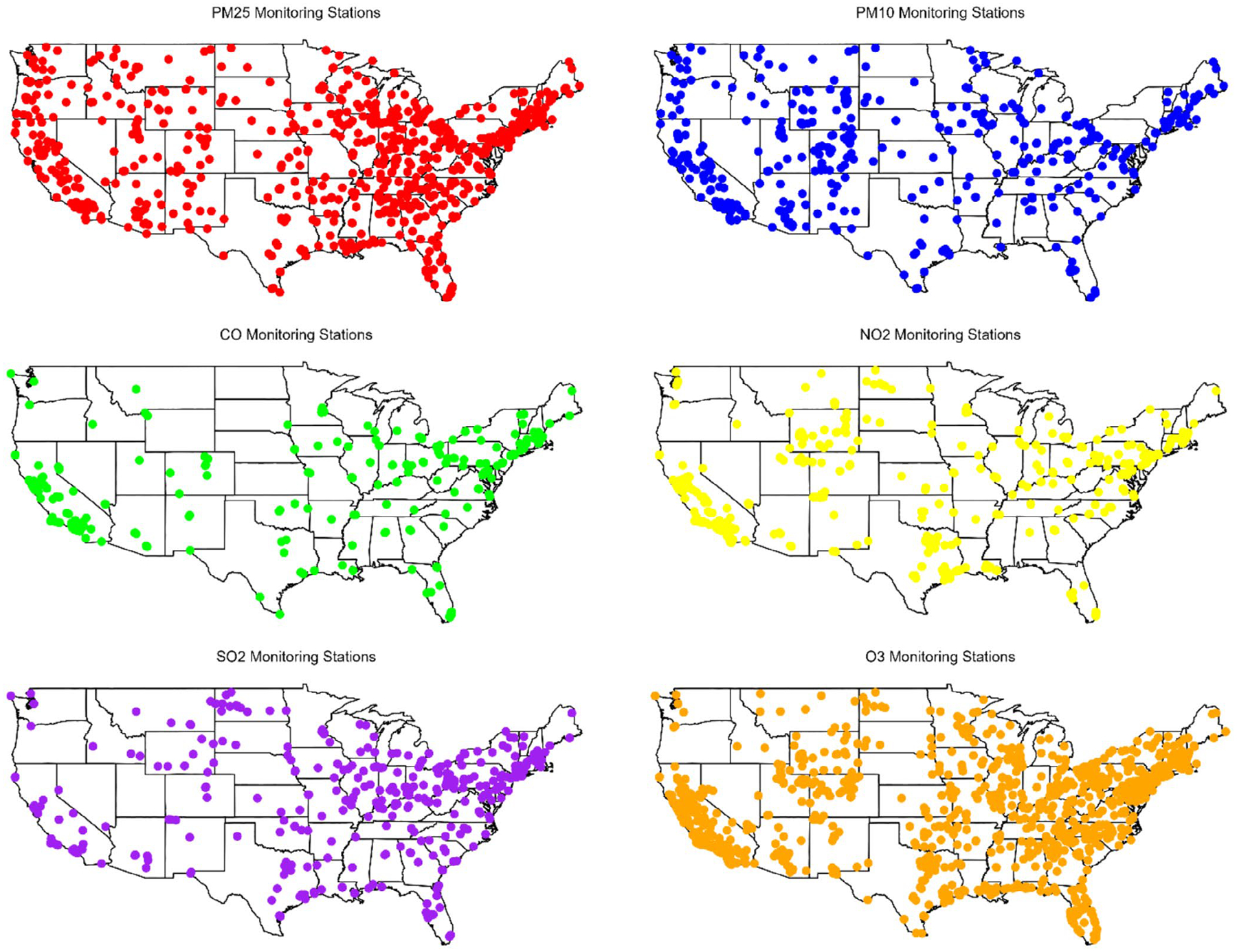
Monitoring stations in the contiguous US in 2016. Each dot on the map represents a US EPA regulatory monitoring station.

The raw data are hourly for gaseous pollutants (NO_2_, SO_2_, CO, O_3_) and daily for particulate matter (PM_2.5_ and PM_10_). We calculated annual-average concentrations of each pollutant (except O_3_) at each site. Selection criteria are (1) a minimum of 18 h of valid measurements per day, to be considered a valid day; (2) at least 244 valid days per year, or at least 61 days for monitors reporting every 3 days, or at least 41 days for those reporting every 6 days (i.e., at least 75% of intended measurements); (3) during the year, a maximum of 45 consecutive days without any measurements (Kim et al. 2020). (The first two criteria reflect EPA criteria. EPA does not have ‘maximum gap’ criteria.) In contrast, for O_3_, the ‘annual average’ metric includes only the heightened O_3_ season from May through September and represents the daily highest 8-h average. Selection criteria for O_3_ are (1) at least 18 h of measurements to be considered a valid day and (2) at least 75% of valid days measured across the period. This process resulted in data collection from a range of 260 to 1089 monitoring stations per pollutant strategically located across various US regions from 2016 to 2020. The breakdown by pollutant was as follows: PM_2.5_ from 495 to 784 stations, PM_10_ from 290 to 454 stations, CO from 228 to 261 stations, NO_2_ from 396 to 414 stations, SO_2_ from 407 to 450 stations, and O_3_ from 1065 to 1089 stations. Figure 1 illustrates the distribution of these monitoring stations. For LUR modelling, annual-average pollutant concentrations were square root transformed to adhere to the normality assumption.

### 2.2 | Independent Data: Geographic and Land Use Features and Satellite Estimates

We used the same predictor variables as in the prior study (Kim et al. 2020). Briefly, the variables cover ten categories: traffic, population, land use/land cover, sources, industrial emissions, vegetation, imperviousness, elevation, position and satellite air pollution estimates (Table 1). These variables aim to capture various anthropogenic and natural factors that may impact air quality. The variables were quantified as count, length, and area within varying buffer sizes ranging from 50 m to 15 km; this approach aims to capture potential local and regional pollution impacts. Satellite-based pollution estimates (i.e., column abundance or surface estimates) are annual averages for NO_2_, PM_2.5_, SO_2_, CO and formaldehyde (HCHO), derived from multiple satellite products (e.g., aerosol optical depth [AOD], ozone monitoring instrument [OMI] and estimates from chemical transport models) (Boersma et al. 2011; Deeter et al. 2017). Consistent with Kim et al. (2020), the HCHO satellite data are a 12-year average (2005–2016) rather than annual averages. HCHO, a significant secondary pollutant formed by the oxidation of VOCs and methane, serves as a valuable indicator of photochemical activity and oxidative capacity in the atmosphere. Its presence may correlate with levels of VOCs and with the formation of secondary pollutants like PM_2.5_ and O_3_, offering crucial insights into overall air quality dynamics. The resolution of the gridded annual satellite-based estimates varied (NO_2_, PM_2.5_, HCHO: 0.1° × 0.1°; SO_2_, CO: 0.25° × 0.25°). Overall, we employ a total of ~360 variables. Additional details regarding data processing and variable calculation are in (Kim et al. 2020).

### 2.3 | Modelling -Building and -Testing

Consistent with previous modelling approaches (Kim et al. 2020; Sampson et al. 2013; Young et al. 2016), we employed a hybrid modelling approach that integrates partial least squares (PLS) with universal kriging (PLS-UK). Specifically, the two components (1) address variance through kriging that utilises an exponential covariance function for the variogram and (2) manage the mean component via PLS that reduces the dimensions of predictor variables in the linear regression process. This combination not only encompasses a broad range of geographic factors but also mitigates the likelihood of generating extreme predictions (Mehmood et al. 2012). Covariance parameters in kriging and regression parameters for PLS summary predictors were selected based on a maximum likelihood approach. We adopted two and three PLS predictors based on cross-validation, respectively, following previous studies using a similar modelling framework (Kim et al. 2020; Young et al. 2016).

To further investigate the model-building process, we employed forward selection to choose an optimal subset of variables from the full set for use in the PLS approach. That is, the process begins by identifying the variable with the highest correlation to the dependent variable and incrementally adding variables that yield the highest partial F-statistic in combination with previously selected variables. This process examines how the number of selected variables influences the model performance.

For model testing, we executed two types of 10-fold cross-validation (CV) training and testing: (1) in conventional CV, monitoring sites are divided into 10 groups randomly; (2) in spatially clustered CV, k-means is used to establish 10 spatial groups (clusters) spatially (Young et al. 2016). In both CV, there are 10 iterations, each involving setting aside one group for model testing and using the remaining groups for model building. Conventional CV evaluates model performance at random locations; spatially clustered CV reflects model performance at sites distant from monitoring stations.

To evaluate model performance via CV comparisons, we utilised standardised root-mean-square error (sRMSE) and a mean squared error (MSE)—based *R*^2^ metric. sRMSE, calculated as RMSE divided by the mean concentrations of all monitors, allows for comparisons across pollutants. MSE-*R*^2^ (hereafter *R*^2^), defined as one minus the ratio of the sum of squared prediction errors to the sum of squared deviations from the observation mean, assesses the fit of the predictions to the 1:1 line rather than the regression line (Knibbs et al. 2016; Wang et al. 2012).

### 2.4 | Model Application

The most effective LUR models identified through this process were then applied to our nationwide model predictions for each pollutant and year. After we identified the best models for each pollutant and respective year, we predicted the annual average concentrations for approximately 7 million Census Block centroids with nonzero population throughout the contiguous U.S. Subsequently, we calculated population-weighted averages at several geographical levels, including Census Block Groups, Census Tracts and Counties for years 2016–2020, aligning with the 2010 Census boundaries. Of the 30 models attempted (i.e., 6 pollutants, 5 years), we were unable to generate one of the models [O3, 2020] because of data constraints, leaving 29 resulting pollutant-year models. This approach was designed to offer our estimates across diverse spatial resolutions for all criteria pollutants, catering to a variety of application needs. All LUR model developments were carried out in R (version 4.2.1).

## 3 | Results and Discussion

### 3.1 | Monitoring Data

**FIGURE 2 |:**
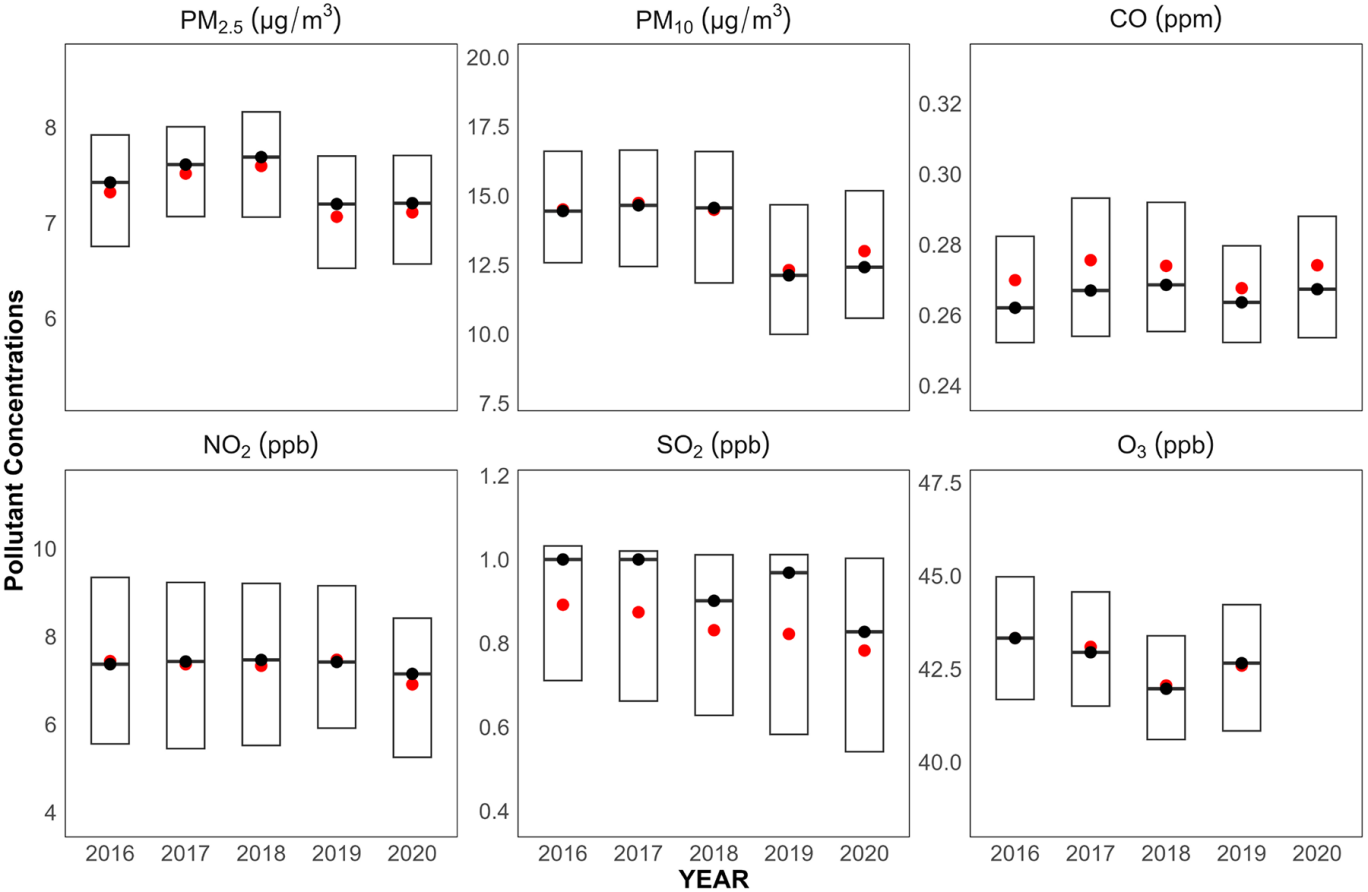
Boxplots of annual average concentrations of six criteria air pollutants across regulatory monitoring sites for 2016–2020 in the contiguous U.S. Each box plot displays the median concentration (black dot) and mean concentration (red dot) and the interquartile range (IQR, represented by the box) of pollutant concentrations for each year. The unit of PM_2.5_ and PM_10_ is μg/m^3^. The unit of CO is ppm. The unit of NO_2_, SO_2_ and O_3_ is ppb.

The main trend for monitoring data we investigated (Figure 2) is, with the exception of O_3_, a slight decline in air pollutant concentrations. This pattern is consistent with the previous years of regulatory monitoring data, indicating improved air quality measures or other environmental policy impacts nationwide (Kim et al. 2020). For PM_2.5_ and PM_10_, the concentrations slightly increased from 2016 to 2018 but decreased in 2019 and 2020. NO_2_ was steady from 2016 to 2019 and showed a slight decrease in concentrations in 2020. SO_2_ generally demonstrated a downward trend in mean concentrations and consistent year-on-year reduction, while CO and O_3_ displayed minor yearly fluctuations with no apparent trend in concentrations.

### 3.2 | Model Evaluation

**FIGURE 3 |:**
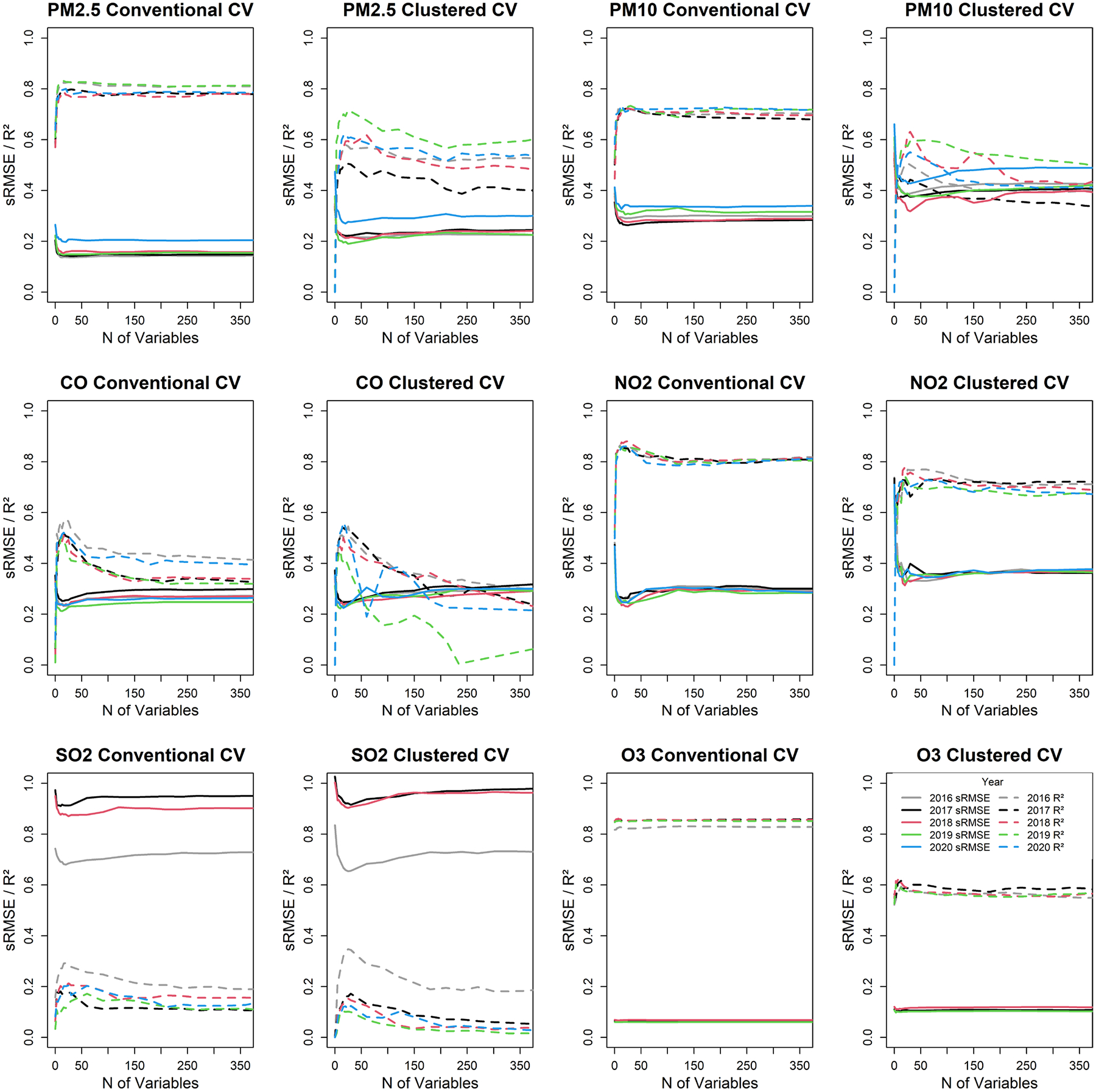
sRMSEs and *R*^2^s of the conventional and clustered CV of LUR models for six criteria air pollutants, 2016–2020, categorised by the number of predictor variables. The solid lines represent sRMSEs, and the dashed lines represent *R*^2^s.

Overall, for each pollutant and across both CV methodologies, LUR models constructed with around 30 predictor variables consistently yielded better *R*^2^ and lower sRMSE as compared to models with more predictors (Figure 3). This pattern underscores the diminishing returns and potential overfitting when too many variables are included (Kerckhoffs et al. 2019; Li et al. 2017). The temporal stability from 2016 to 2020 of model performance also highlights the robustness of the LUR models. These results imply that a more parsimonious approach to model development, that is, selecting only the most pertinent categories of predictor variables, can provide more reliable models; that finding is consistent with prior research (Kim et al. 2020; Meng et al. 2015).

In terms of the two CV approaches, LUR models validated using the conventional CV method consistently outperformed those using the spatially clustered CV approach, as indicated by higher *R*^2^ and lower sRMSE values (Figure 3). That result is consistent with expectations and with the previous modelling effort (Kim et al. 2020). As mentioned above, clustered CV reflects model performance far from other monitors.

**FIGURE 4 |:**
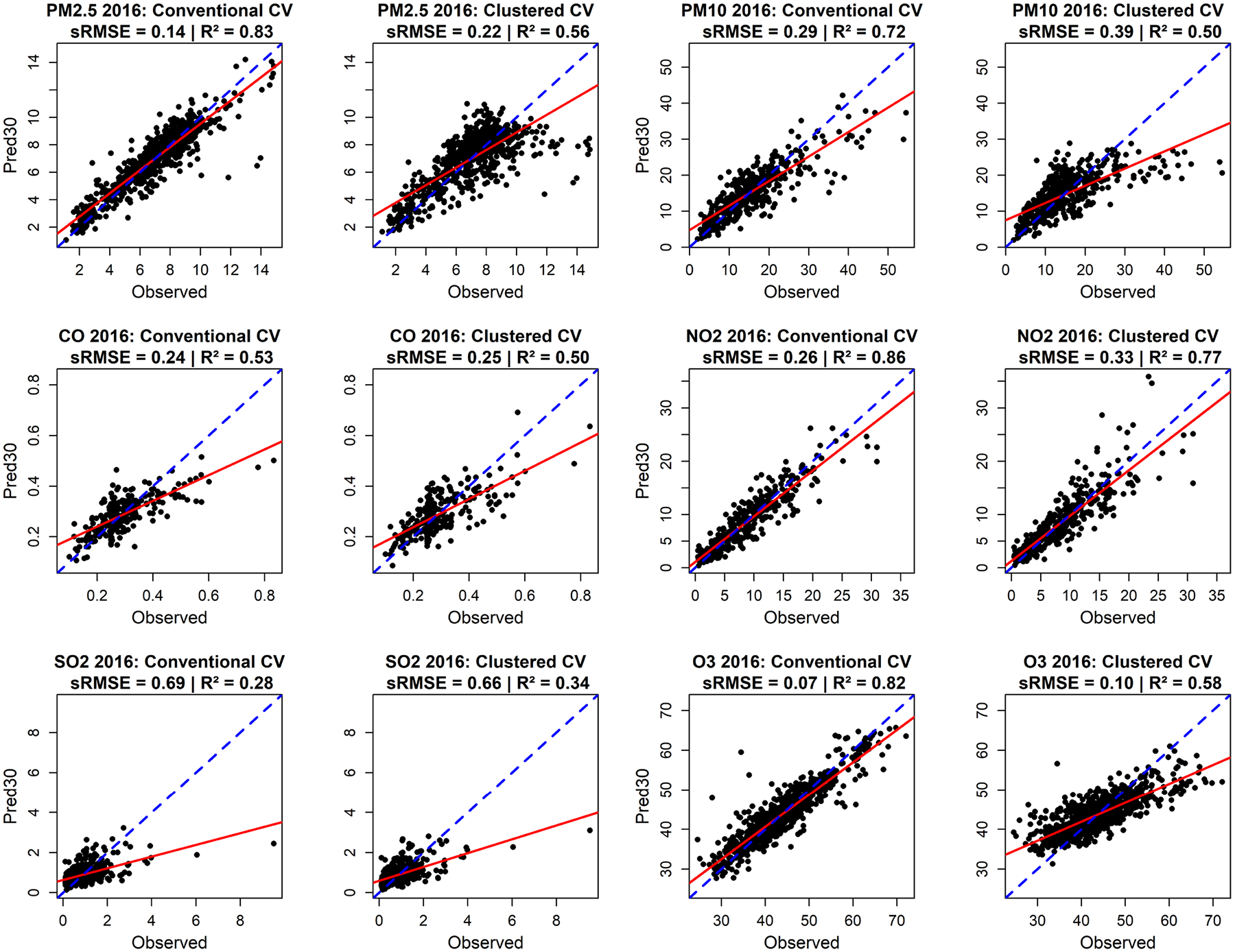
Scatterplots of the sRMSEs and *R*^2^s of the conventional and clustered CV of LUR models using the ‘best’ number of predictor variables for six criteria air pollutants in 2016. Green line indicates the 1:1 line and red line indicates the fitted line.

Table 2 shows the performance of LUR models using the ‘best’ number of predictor variables. In general, model performance is consistently higher for PM_2.5_, PM_10_, NO_2_ and O_3_ than for CO and SO_2_, particularly when using conventional CV. Over the five-year span, this trend is evident through the sustained *R*^2^ values above 0.70 for PM_2.5_ and PM_10_ and exceeding 0.80 for NO_2_ and O_3_, underscoring the robustness of the LUR models for these pollutants. Scatterplots of the LUR models using the ‘best’ number of predictor variables for six criteria air pollutants in 2016 (Figure 4) illustrate the level of agreement between predicted versus measured air pollution levels. Scatterplots reveal similar patterns as in Table 2.

### 3.3 | Model Application

After model-building and -testing, we applied the model across geographies, estimating concentrations at each Census Block and then population-weighted averaging to the Block Group (year-2016: Figure 5), tract, and county. The maps revealed the heterogeneous distribution of each air pollutant. As expected, the PM_2.5_ concentrations were notably higher in California and along some urbanised areas of the East Coast, reflecting the combined impact of energy production and dense population centres (Kelly et al. 2021). PM_10_ levels showed similar patterns, likely also reflecting the influence of both natural desert dust and anthropogenic sources (Chen et al. 2010). SO_2_ levels showed slightly higher levels in Southern and Central states, potentially near industrial sources (Nopmongcol et al. 2019). O_3_ concentrations demonstrated a broad distribution, with higher levels in the Southern and Southwestern states, which could be attributed to these regions’ warmer climates, conducive to O_3_ formation (Jiao et al. 2016). These maps underscore the diverse spatial patterns of air pollutant concentrations across the U.S., reflecting regional differences in emission sources, environmental policies and natural factors.

The summary statistics for population-weighted annual average concentrations of air pollutants from 2016 to 2020 revealed an upward trend in PM_2.5_ exposure, with both median and mean concentrations rising over the five-year period (Table 3). In contrast, NO_2_ and SO_2_ levels decreased, suggesting improvements in these pollutants nationwide. PM_10_ and O_3_ concentrations fluctuated year to year without a clear long-term trend, indicating variability in exposure to these pollutants across the U.S. population.

### 3.4 | Comparison With the Previously Developed Database (1979–2015)

The consistent methodology across the current and historical datasets enables users to track long-term trends in air pollution exposure with confidence. Notably, the trend of decreasing NO_2_ and SO_2_ levels aligns with historical data, suggesting ongoing improvements in air quality. However, the recent increase in PM_2.5_ and PM_10_ concentrations as observed in our updated models disagreed with the previous models’ decreasing trend. This finding is consistent with some recent studies (Bekbulat et al. 2021), which warrant further investigation into the sources of particulate pollution. The variability observed in the O_3_ concentrations is consistent with the historical dataset (Kim et al. 2020). The prediction maps further emphasised the spatial consistency in air pollution distribution as compared to previous periods. For example, areas historically impacted by higher concentrations of PM_2.5_ and PM_10_, such as California, continue to show elevated levels, while regions with industrial activities remain hotspots for SO_2_. These spatial and temporal trends of consistency highlight the persistent influence of both anthropogenic activities and natural impacts on ambient air quality.

### 3.5 | Dataset Location and Format

Model predictions can be freely downloaded online at https://www.caces.us/data. The available dataset provides population-weighted annual-average concentrations for the six criteria air pollutants at three geographic levels (Census Block Groups; Census Tracts; Counties) during 2016–2020 (as noted above, O_3_ estimates are for summer months and estimates for 2020 are unavailable).

### 3.6 | Limitations and Future Work

Our comprehensive high-resolution geospatial database supplements the previously developed air pollution database (1979–2015) and provides detailed estimates of population-weighted annual-average concentrations for the six criteria pollutants (PM_2.5_, PM_10_, CO, NO_2_, SO_2_, O_3_) across varying geographic scales, spanning the combined years 1979–2020.

While our model provides comprehensive geospatial estimates of air pollutant concentrations, it is not without limitations. The reliance on regulatory monitoring data can lead to underrepresentation of air quality in regions with sparse monitoring infrastructure, potentially affecting the precision of estimates in rural or remote areas (deSouza and Kinney 2021; Kelp et al. 2022). Future enhancements could include the integration of other remote sensing products (e.g., Google Earth Engine) and low-cost sensor networks (e.g., PurpleAir) to increase spatial coverage, especially in under-monitored regions (Brook et al. 2018; Lee et al. 2017; Lu et al. 2022). Modelling performance is not as strong for CO and SO_2_ as for other pollutants. That result may reflect several possible aspects, including the number and placement of monitors, spatial patterns, and the degree to which those patterns correlate with land-use or other data used in our model. Future research might incorporate more detailed emission inventory data into the model development, aiming to capture point sources and industrial emissions that could significantly affect local concentrations. Our current modelling framework is PLS-UK, which aims to maintain methodological consistency with previous studies and allows for direct comparisons over time; future modelling approaches could include other geospatial and empirical models (e.g., machine learning, geographically weighted regression) (Lloyd et al. 2023; Lotfata 2022; Lu et al. 2021). Furthermore, the temporal resolution here is confined to annual averages, which incorporates but does not shed light on short-term variability, episodic events or seasonal patterns (Dons et al. 2014); future work could advance the temporal resolution of our models to capture temporal dynamics; this approach may, potentially, inform timely and targeted air quality interventions (Gladson et al. 2022; Masiol et al. 2018; Yuan et al. 2022). To be consistent with the prior modelling efforts (Kim et al. 2020), our approach is based on regulatory monitors and so is confined to criteria pollutants; future work could consider air toxics or emerging pollutants, if monitoring data were available (Amini et al. 2017; Jedynska et al. 2014; Saha et al. 2021).

## 4 | Conclusion

We developed a high-resolution geospatial database consisting of the population-weighted annual average concentrations of six key criteria air pollutants across the contiguous U.S. from 2016 to 2020, at several geographical levels, including Census Block Groups, Census Tracts and Counties. Our models were developed using a PLS-UK framework; the models achieved reasonable performance, consistent with our earlier published models.

## Figures and Tables

**FIGURE 5 |:**
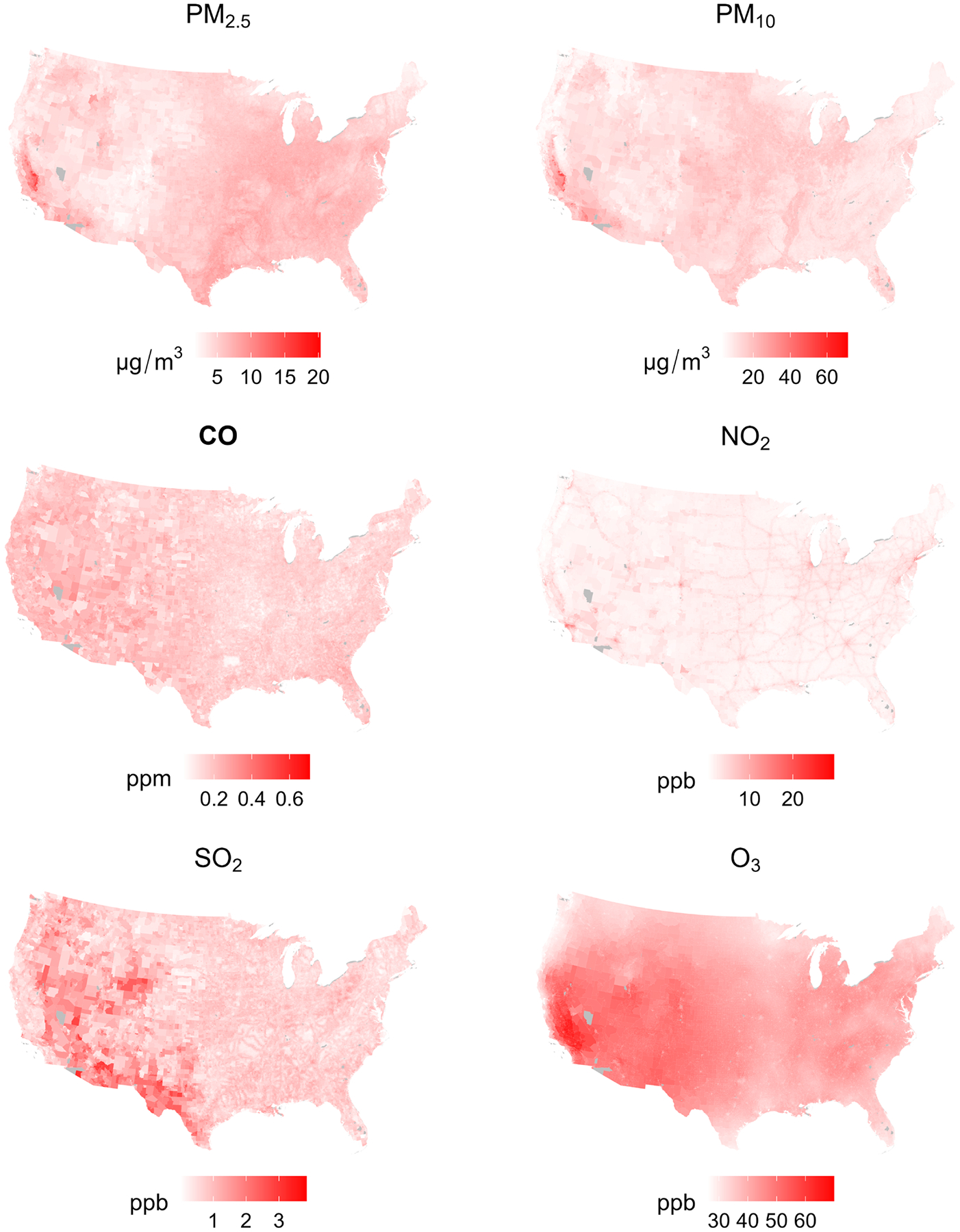
Maps of Census Block Group population-weighted annual average concentrations for six criteria air pollutants in 2016 based on the ‘best’ LUR models.

**TABLE 1 |:** List of geographic and land use variables and satellite estimates used in the LUR models.

Category	Measure	Spatial resolution	Description	Data source^a^
Traffic	Length/density in buffer (km)	0.05–15 km	Any road, truck route, intersections, etc	TeleAtlas
Population	Count in buffer	Block group	Population in block groups (0.5–3 km)	U.S. Census
Land use/land cover	Area in buffer (%)	30 m	Built land, open space, agricultural land, etc. (0.05–15 km)	NLCD
Sources	Length in buffer (m)	Point	Distance to the nearest source (e.g., railroad, airport)	NEI
Emissions	Point in buffer (ton)	Point	Sum of site-specific facility emissions (3–30 km)	NEI
Vegetation	Area in buffer	30 m	Normalised difference vegetation index (0.5–10 km)	University of Maryland
Impervious	Area in buffer (%)	30 m	Impervious surface value (0.05–5 km)	NLCD
Elevation	Value	30 m	Elevation above sea levels; counts of points above or below a threshold (1–5 km)	USGS NED
Position	Coordinates	Point	Latitude, longitude	EPA
Satellite estimates	Column abundance or surface (μg/m^3^ or ppb)	10–25 km	Satellite-based air pollution estimates (NO_2_, SO_2_, CO, HCHO, PM_2.5_)	Multiple sources^a^

*Note:* Detailed information about the data sources can be found in Kim et al. 2020 and Lu et al. 2021. TeleAtlas provides dynamic location-based data. NLCD is the National Land Cover Database; NEI is the National Emission Inventory; USGS NED is the National Elevation Dataset (NED) of the U.S. Geological Survey (USGS).

a Satellite-derived annual average estimates for PM_2.5_ (1998–2014, 0.1° * 0.1° grid), NO_2_ (2004–2015; 0.1° * 0.1° grid), SO_2_ (2005–2016; 0.25° * 0.25° grid) and CO (2001–2016; 0.25° * 0.25° grid).

**TABLE 2 |:** sRMSEs and *R*^2^s of the conventional and clustered CV of LUR models using the ‘best’ number of predictor variables for six criteria air pollutants, 2016–2020.

		Conventional CV		Clustered CV	
Pollutant	Year	*R* ^2^	sRMSE	*R* ^2^	sRMSE
PM_2.5_	2016	0.83	0.14	0.56	0.22
PM_2.5_	2017	0.80	0.14	0.5	0.22
PM_2.5_	2018	0.77	0.16	0.58	0.22
PM_2.5_	2019	0.83	0.15	0.71	0.19
PM_2.5_	2020	0.78	0.21	0.61	0.28
PM_10_	2016	0.72	0.29	0.50	0.39
PM_10_	2017	0.72	0.27	0.44	0.37
PM_10_	2018	0.72	0.28	0.63	0.32
PM_10_	2019	0.73	0.31	0.59	0.38
PM_10_	2020	0.72	0.34	0.55	0.43
CO	2016	0.53	0.24	0.50	0.25
CO	2017	0.48	0.26	0.53	0.25
CO	2018	0.44	0.25	0.45	0.25
CO	2019	0.41	0.23	0.36	0.24
CO	2020	0.49	0.24	0.48	0.25
NO_2_	2016	0.86	0.26	0.77	0.33
NO_2_	2017	0.83	0.28	0.66	0.40
NO_2_	2018	0.87	0.24	0.76	0.33
NO_2_	2019	0.86	0.24	0.70	0.35
NO_2_	2020	0.85	0.26	0.70	0.36
SO_2_	2016	0.28	0.69	0.34	0.66
SO_2_	2017	0.17	0.91	0.17	0.92
SO_2_	2018	0.20	0.88	0.15	0.91
SO_2_	2019	0.14	1.65	0.10	1.69
SO_2_	2020	0.17	1.45	0.12	1.49
O_3_	2016	0.82	0.07	0.58	0.10
O_3_	2017	0.85	0.06	0.60	0.11
O_3_	2018	0.85	0.07	0.58	0.12
O_3_	2019	0.85	0.06	0.57	0.10

*Note:* sRMSE, calculated as RMSE divided by the mean concentrations of all monitors. *R*^2^ represents MSE-*R*^2^, defined as one minus the ratio of the sum of squared prediction errors to the sum of squared deviations from the observation mean, and it assesses the fit of the predictions to the 1:1 line rather than the regression line. In conventional CV, monitoring sites are divided into 10 groups randomly; in spatially clustered CV, k-means is used to establish 10 spatial groups (clusters) spatially (Young et al. 2016).

**TABLE 3 |:** Summary statistics^a^ of population-weighted annual average concentrations^b^ across all Census Block Groups for the contiguous U.S., 2016–2020, based on the ‘best’ LUR models.

Pollutant	Year	P10	P25	Median	P75	P90	Mean	SD
PM_2.5_	2016	5.49	6.43	7.49	8.27	9.07	7.40	1.56
PM_2.5_	2017	5.75	6.68	7.61	8.48	9.39	7.64	1.60
PM_2.5_	2018	5.80	6.69	7.70	8.72	10.05	7.86	1.82
PM_2.5_	2019	5.14	6.15	7.18	8.03	8.93	7.10	1.55
PM_2.5_	2020	5.27	6.17	7.19	8.33	10.00	7.55	2.37
PM_10_	2016	10.51	12.94	16.06	19.38	22.92	16.70	5.63
PM_10_	2017	10.64	13.03	15.82	19.44	22.99	16.71	5.65
PM_10_	2018	10.71	13.03	15.99	19.87	24.07	16.93	5.66
PM_10_	2019	9.33	11.71	14.54	17.91	21.05	15.09	4.99
PM_10_	2020	9.02	12.01	15.95	20.86	26.37	16.97	7.04
CO	2016	0.16	0.19	0.23	0.27	0.31	0.23	0.06
CO	2017	0.17	0.19	0.22	0.27	0.32	0.23	0.07
CO	2018	0.17	0.19	0.23	0.27	0.31	0.23	0.06
CO	2019	0.15	0.19	0.22	0.25	0.29	0.22	0.05
CO	2020	0.16	0.19	0.22	0.26	0.30	0.23	0.06
NO_2_	2016	2.31	3.61	5.97	8.71	12.72	6.81	4.23
NO_2_	2017	2.22	3.24	5.33	8.22	12.49	6.43	4.31
NO_2_	2018	2.23	3.43	5.54	8.07	11.93	6.38	3.99
NO_2_	2019	2.29	3.49	5.66	8.27	11.80	6.43	3.85
NO_2_	2020	1.94	3.19	5.16	7.57	10.91	5.83	3.57
SO_2_	2016	0.48	0.60	0.76	0.94	1.13	0.79	0.28
SO_2_	2017	0.38	0.52	0.68	0.85	1.04	0.70	0.28
SO_2_	2018	0.34	0.47	0.62	0.80	0.99	0.65	0.27
SO_2_	2019	0.26	0.40	0.59	0.82	1.04	0.63	0.32
SO_2_	2020	0.24	0.38	0.55	0.73	0.91	0.57	0.27
O_3_	2016	34.81	39.22	42.48	44.64	48.08	42.12	5.69
O_3_	2017	35.24	39.00	41.88	44.46	50.05	42.24	6.16
O_3_	2018	34.66	37.81	40.75	42.73	49.83	41.15	5.94
O_3_	2019	35.03	38.35	41.55	44.08	48.71	41.61	5.68

Abbreviations: SD, standard deviation.

a P10 represents the 10th percentile. P25 represents the 25th percentile. P75 represents the 75th percentile. P90 represents the 90th percentile.

b The unit on PM_2.5_ and PM_10_ is μg/m^3^. The unit on CO is ppm. The unit on NO_2_, SO_2_ and O_3_ is ppb.

## Data Availability

The high-resolution geographic database (estimated ambient annual-average concentrations) can be downloaded as CSV files freely from the official website of the Center for Air, Climate, and Energy Solutions (CACES): https://www.caces.us/data.
